# Watermelons, Syringes, and Regulation: Instagram Marketing by Cosmetic Clinics in the UK and the Netherlands

**DOI:** 10.1007/s00266-023-03420-0

**Published:** 2023-06-19

**Authors:** Anne-Mette Hermans

**Affiliations:** 1 Tilburg School of Social and Behavioral Sciences, Tilburg University, Warandelaan 2, 5037 AB Tilburg, The Netherlands; 2 Erasmus University Rotterdam, Burgemeester Oudlaan 50, 3062 PA Rotterdam, The Netherlands

**Keywords:** Cosmetic procedures, Social media, Marketing, Regulation, Ethics

## Abstract

**Background:**

Cosmetic clinics use social media to inform and market to prospective patients. Evidence from social media posts by the UK and Dutch cosmetic clinics illustrates the precarious balance between professionalism and commercialism, and raises important questions for the regulation of these marketing communication efforts.

**Methods:**

A random selection of Instagram posts by Dutch and the UK clinics which offer (non-) invasive cosmetic procedures were selected for a qualitative content analysis. The corpus of data comprised 395 posts by six Dutch and four UK clinics, published between January 2018 and July 2019. The method of analysis was inspired by previous qualitative studies into the marketing of cosmetic procedures and can be described as a (discursive) thematic analysis.

**Results and Conclusions:**

The tension between the medical-professional and commercial nature of cosmetic procedures was evident in the Instagram posts by Dutch and UK clinics. Despite calls for ‘ethical’ marketing on social media, this study illustrates that marketing materials are not always in line with current advertising guidelines. Whereas advertising standards authorities have warned against the trivialization of cosmetic procedures, posts by clinics backgrounded the medical nature of procedures in favor of more commercial advertising appeals. Furthermore, the posts demonstrated little diversity in terms of models’ gender, ethnicity and body type, which reinforces narrow contemporary beauty ideals.

**Level of Evidence V:**

This journal requires that authors assign a level of evidence to each article. For a full description of these Evidence-Based Medicine ratings, please refer to the Table of Contents or the online Instructions to Authors www.springer.com/00266.

## Introduction

In line with changing media and advertising landscapes, cosmetic providers are turning to social media to market their services [1]. As previous studies have indicated, recipients of cosmetic procedures are likely to consult social media for information and recommendations before undergoing procedures [2, 3]. Although the importance of social media has been acknowledged for cosmetic practitioners and clinics, few studies have analyzed the contents that these providers publish on social media platforms [4]; rather, previous research has predominantly focused on the (reputability of the) information source behind the content [4, 5]. So, to address the need to understand the content of these social media posts, this paper was guided by the following research question: “how do cosmetic clinics in the Netherlands and the United Kingdom present themselves on Instagram?” To address this question, several foci were included, namely the construction/reinforcement of beauty (ideals) by clinics; tensions between the medical/professional and the commercial sides of the cosmetic surgery industry [3]; affordances of social media marketing; and, finally, the various ethical and regulatory concerns surrounding the marketing of cosmetic procedures [6].

Both the Netherlands and the UK rank within the global top 30 in terms of the estimated number of plastic surgeons [7]. Moreover, specific advertising guidelines related to the marketing of cosmetic procedures are in effect in both countries [8–10]. Nevertheless, the normalization and regulation of the industry differs; in the Netherlands, for example, the title ‘cosmetic doctor’—referring to medical doctors who offer noninvasive cosmetic treatments like injectables—is protected and requires a two-year additional training course [11]. The UK, on the other hand, is still debating a stricter ‘licensing regime’ for noninvasive cosmetic procedures [12].

### Marketing Cosmetic Procedures

Previous research examined cosmetic surgery advertising in various types of traditional media, such as city magazines, men’s and women’s lifestyle magazines, and advertising leaflets [13, 14]. These studies generally found that advertising materials include some practical information regarding the clinics, procedures, and the costs of these procedures; however, potential side effects, recovery time, or risks were rarely mentioned [15]. Moreover, Lirola and Chovanec [14], for example, found that procedures were presented as quick and easy solutions to improve both physical and psychological aspects of the body and self. Generally, marketing for cosmetic procedures has been shown to background, or even disregard, the medical reality of procedures in favor of messages related to the positive physical and psychological results of procedures.

Reflecting neoliberal climates in which individualism and personal agency are fundamental, marketing for cosmetic procedures reflects and sells these values as it emphasizes and lauds self-determination [15, 16]. People are constructed as individually responsible for the health and appearance of their bodies, which have become objects and projects [17–19]. This conceptualization of bodies is reinforced by consumer culture as body projects require people to engage in constant self-examination and improvement through consumption [20].

Acknowledging the increased importance of social media platforms for the representation and marketing of cosmetic procedures, more recent research has focused on issues of both the content creators behind—and at times the contents of—these social media messages [1–3]. Generally, these studies confirm previous findings as information regarding the nature of the medical procedures and/or potential complications remains underrepresented [4, 21, 22].

Although cosmetic procedures are inherently medical, their elective nature and predominant focus on improving bodily appearance rather than function sets them apart from other medical procedures^1^. This unique positioning of cosmetic procedures is reflected in the apparent tension between professionalism and commercialism [3], or education and promotion [1], in contemporary marketing materials for cosmetic procedures. This tension has been explored through the positioning theory framework, which postulates that actors present themselves in a particular way to realize certain outcomes; medical professionals, for example, may adopt the position of ‘guardian of patients’ health’ or of ‘self-interested businessperson with skills to sell’ [4].

#### Advertising Regulations and Ethics

The (ethical) nature of marketing for cosmetic procedures has been discussed widely; Sullivan ([49], pp. 409–410), for example, posited that the practice of influencing people’s decisions to undergo cosmetic procedures—particularly by the physicians “entrusted by a patient to evaluate the chance of doing good against the possibility of doing harm”—raises significant ethical issues, especially considering the significant role that advertising for cosmetic procedures can play in normalizing procedures, and how it positively influences people’s cosmetic surgery acceptance and intention [23, 24]. Sharing these concerns, countries across the world (e.g., Germany, the UK, France, and China) have proposed strict regulations—or even a ban—on cosmetic surgery advertisements, particularly those aimed at young people [25–27]. Nevertheless, particularly regulation for online marketing has proven difficult as this requires a uniform, international approach [28].

Both the Netherlands and the UK have regulatory bodies which developed specific guidelines for the advertising of cosmetic procedures. The UK’s Advertising Standards Authority (ASA) developed the Advertising Guidance on Cosmetic Interventions (2016, 2021). This Guidance is critical of the trivialization of cosmetic procedures, as it stipulates that advertising cannot suggest that cosmetic procedures “can be undertaken lightly” [9] and that marketers must not “imply that invasive surgery is a ‘minor procedure’ or similar if that claim is likely to mislead as to the complexity or duration of the operation, the pain experienced either during or after the operation, the length of the recovery time, or the potential side-effects”. Moreover, the Guidance aims to protect young people as it prohibits marketing for cosmetic procedures directed at under-18s either “through the selection of media or context in which they appear”. Although the ASA is reactive and has little power to enforce these guidelines, it can refer cases to the UK’s communications regulator, the Office of Communications (Ofcom), or to Trading Standards [28].

In the Netherlands, the Stichting Reclame Code [Foundation Advertising Code] published a specific Code for medical cosmetic treatments performed by doctors (CCBA) in 2018. Like the ASA’s guidelines, the Code stipulates that advertising must be objective, cannot mislead, and procedures should not be trivialized. In line with this, the Code prohibits any marketing communication “which does not promote the rational use of a medical cosmetic treatment” and requires that images must be “truthful” [my translations] [10]. Furthermore, acknowledging the detrimental impact of viewing narrow body ideals in traditional and social media on people’s body perception and self-image [29], marketing for cosmetic procedures cannot “give the impression that a particular body shape or specific physical characteristic is preferred” [my translation] [10]. As will be demonstrated below, this latter rule may be problematic as the practice of cosmetic procedures can propagate and confirm a particular beauty standard.

## Materials and Method

To answer the research question of how cosmetic clinics present themselves on Instagram, I conducted a qualitative content analysis of 701 Instagram posts by various Dutch and the UK clinics offering (non-) invasive cosmetic procedures. These clinics were selected via random sampling and snowball sampling. As can be seen from Table 1, there were important differences between the clinics; on average, the UK clinics produced a greater number of posts (*M* = 2734) and had more followers (*M* = 30.825) when compared to Dutch clinics (*M*_posts_ = 425; *M*_followers_ = 10.404). Moreover, the clinics offer different cosmetic procedures; the Dutch Alizonne and Van Rosmalen clinics, for example, only offer noninvasive procedures such as injectables and laser treatments, whereas The Harley Medical Group specializes solely in invasive, surgical procedures. Nevertheless, the majority of the clinics offer both invasive and noninvasive procedures.

**Table 1:** Overview clinics NL and the UK (correct as of 11/10/2022)

Clinics	No. of posts	No. of followers	First post on IG
*The NL*			
Aklinieken	573	4333	26/01/2016
Alizonne	41	4809	22/12/2017
Faceland Nederland	302	40.600	10/08/2017
Van Rosmalen Kliniek	597	5774	13/09/2014
Kliniek Veldhoven	315	1767	5/12/2016
Velthuis Kliniek	722	5140	24/01/2017
*The UK*			
Harley Medical Group	463	13.500	21/09/2016
Linia Cosmetic Surgery	1276	10.100	01/08/2016
MYA Cosmetic Surgery	7661	72.200	11/09/2015
Transform Hospital Group	1539	27.500	01/10/2015

To compile the corpus, I collected a random sample of posts published between the 1^st^ of January 2018 and the 1^st^ of July 2019. Any post could be selected as I aimed to obtain a representative idea of how cosmetic clinics present themselves on Instagram. In total, I analyzed 395 posts by the six Dutch clinics and 306 posts by the four UK clinics. As Instagram posts constitute ‘holistic units’ consisting of images/videos, text, emojis, and hashtags, these were all considered [30].

The method of analysis was inspired by previous qualitative studies into the marketing of cosmetic procedures and can be described as a (discursive) thematic analysis [13, 20, 31]. As thematic analysis neither entails nor prescribes just one method of data analysis, nor does it provide concrete or rigid ‘rules’, it is important to discuss the adopted approach and the tools which aided the analytical process [32]. For this research project, previous research into the marketing of medical cosmetic procedures provided a lens through which I engaged with the data; in this sense, the coding was deductive. Moreover, to explore whether clinics presented narrow body ideals, I coded the models’ ethnicity and body shape. As I wanted to assess whether the thin-/fit-ideal for women and the muscular ideal for men was reinforced by the clinics’ posts, I coded models’ level of muscularity (i.e., low, moderate, high, and extreme) and, in line with De Freitas, Jordan and Hughes [33], I adopted Stunkard et al.’s (1983) Figure Rating Scale to code the models’ bodies, indicating whether the models were underweight (FRS 1-2), appropriate weight (FRS of 3-4), slightly overweight (FRS 5), overweight (6–7), or obese (FRS 8–9)^2^ [2, 23].

Reflecting the qualitative nature of this project, I remained sensitive to elements in the data which provided additional or alternative insights related to how cosmetic clinics market themselves on Instagram in particular. This form of thematic analysis can be described as ‘codebook’ TA, where the researcher generates themes early on and uses “some kind of structured coding framework for developing and documenting the analysis” [32]. Despite the early development of themes, however, these can be refined and “new themes can be developed through inductive data engagement and the analytic process” (*ibid*).

## Results and Discussion

This section presents the main findings of the content analysis of the cosmetic clinics’ Instagram posts. Firstly, it is necessary to explore the clinics’ reinforcement of contemporary beauty ideals for both men and women. Following this, the previously established tension between professionalism and commercialism in marketing by cosmetic clinics is discussed. The clinics included discourses related to the medical nature of cosmetic procedures, but mostly centered around the positive physical and mental outcomes of these procedures, for example highlighting recipients’ attractive physiques and/or the accompanying increase in self-confidence [3, 4, 15]. Lastly, the clinics’ use of humor and emojis are explored as these may contribute to a lighthearted framing of cosmetic procedures.

### Marketing the White, Slim Body

As approximately 90% of cosmetic procedures are undertaken by women [7, 34], it is unsurprising that most models shown in the posts by cosmetic clinics were female (i.e., 89% [*n* = 236] of models in Dutch posts and 98% [*n* = 265] in the UK posts). The few posts containing male models emphasized the acceptability and (increasing) normality of cosmetic procedures for men, echoing Davis’ (2002) observation that media portrayals of men undergoing cosmetic procedures demonstrate an awareness of their relative untypicality [35]. Challenging the prevalent idea that cosmetic procedures would just be for women, a post by MYA (June 30, 2019), for example, stated, “men have cosmetic surgery too, it doesn’t make them any less manly or vain, it’s a personal choice just like it is for women”.

Despite calls to diversify bodies and appearances in the media and body positivity movements which challenge and reject narrow thin- and/or fit ideals in favor of more diverse and inclusive body representations [36, 37], the models in the clinics’ posts displayed little ethnic or body diversity. The men and women in both the Dutch and the UK posts were overwhelmingly white (92%, *n* = 216 and 86%, *n* = 229, respectively), which echoes findings by previous research [15, 20]. Although white women have been shown to hold more positive attitudes toward, and are more likely to undergo, cosmetic procedures when compared to people from ethnic minorities [38, 39], this overrepresentation of white models may also construct and reinforce the problematic notion of ‘whiteness’ as a desirable appearance characteristic.

**Fig. 1:**
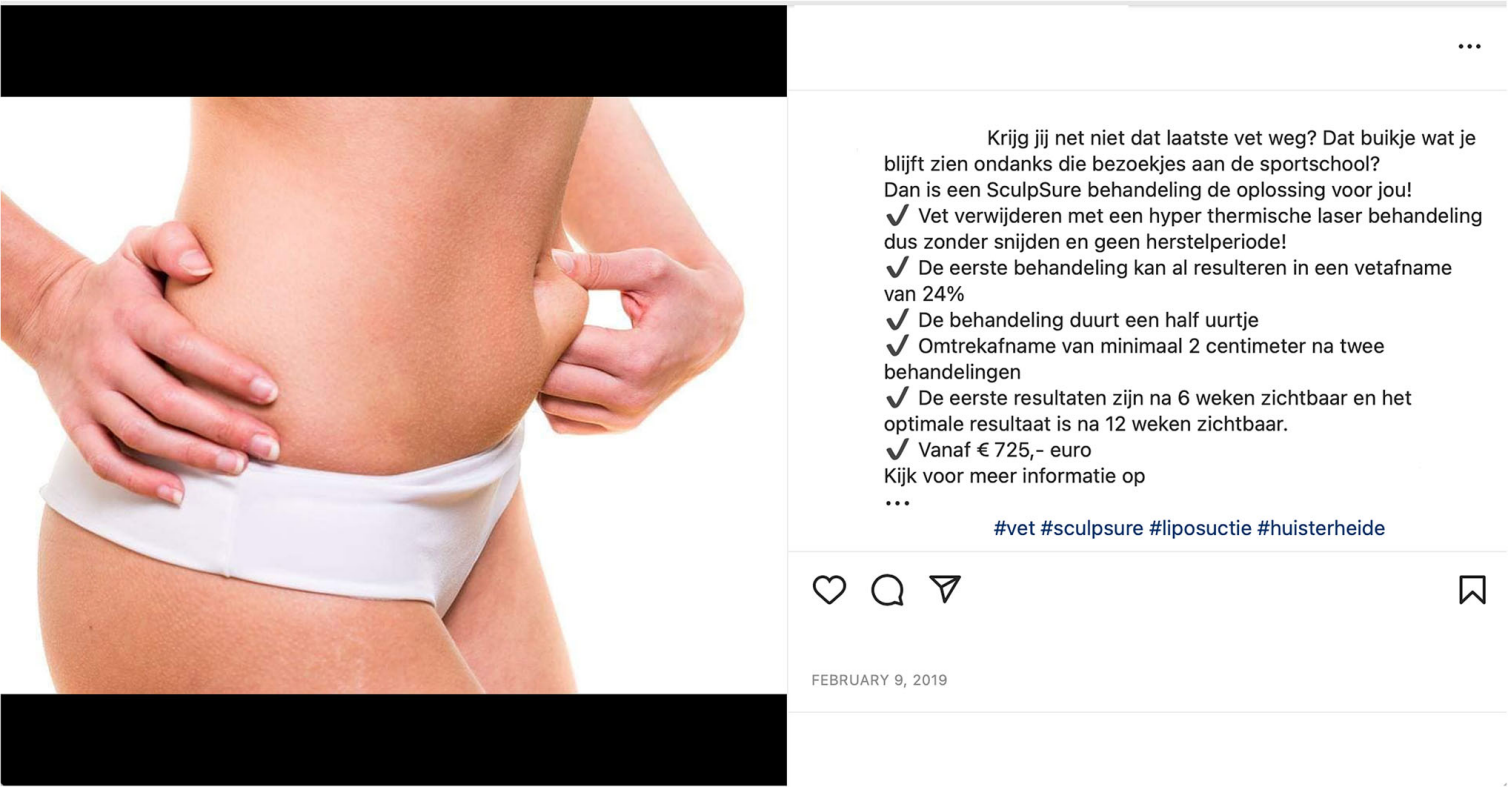
Can you not get rid of that last bit of fat? That belly bulge you keep seeing despite those gym visits? Then a SculpSure treatment is the solution for you!

In terms of body shape, the female models displayed low to moderate muscularity and had an average FRS of 3.4, which means they would have an ‘appropriate’ weight when adopting the classification by De Freitas, Jordan and Hughes [33]. Although the cosmetic clinics seem to avoid a direct glorification of (unhealthily) thin bodies, they do problematize and medicalize bodies which are larger than the ‘appropriate’ weight norm. In the posts featuring female models coded as (slightly) overweight, 45% [*n* = 5] promoted various weight-related procedures, like (vaser) liposuction; moreover, all eight posts including ‘overweight’ or ‘obese’ models (FRS ratings of 6+) endorsed weight loss procedures. The images of ‘overweight’ models were often taken before a procedure and were accompanied by ‘after’ images in which models had achieved an ‘appropriate’ weight (FRS 3-4), thereby reinforcing the message that the overweight body is problematic and needs to be ‘corrected’ by cosmetic procedures to be in-line with normative societal ideals. Models with an ‘appropriate’ weight were at times also used to market fat removal procedures; in a post for laser fat removal, for example, a woman is shown to be pinching ‘fat’ on her stomach, accompanied by the caption asking followers whether they recognize the struggle to get rid of “that last little bit of fat”, or the gym-resistant “slight belly bulge” (my translation; see Fig. 1).

The few male models included by the cosmetic clinics displayed moderate to high levels of muscularity and had a higher FRS (i.e., 4.7) than the female models, which is unsurprising considering male body ideals in the UK and the Netherlands. Nevertheless, similarly to the framing of the (slightly) overweight female models, three out of four male models who were coded as (slightly) overweight were used to promote weight loss procedures like liposuction. In general, then, clinics across the UK and the Netherlands can be seen to reinforce a narrow contemporary body ideal: slim and white for women, and slim, yet muscular, and white for men.

### Professionalism: Medical aspects

Although cosmetic procedures are at the core of the clinics’ offerings, just 60% [*n* = 236] of the posts by Dutch clinics and approximately half (52%, *n* = 158) of the UK clinics referred to cosmetic procedures. In line with previous research which found that the medical nature of cosmetic procedures was generally minimized in cosmetic surgery advertising, few Instagram posts by the clinics reflected medicality in their visuals and/or captions [4, 6, 15]. Visually, the clinics signaled medicality in several ways; for example, 12% [*n* = 49] of the Dutch clinics and 7% [*n* = 22] of the UK clinics included medical professionals. Interestingly, the Dutch medical professionals were significantly more frequently shown in medical attire than their UK counterparts (χ^2^[1, 1]=13.23, *p*=.003), conveying a sense of authority. The inclusion of these medical figures could be considered part of what Hennink-Kaminski, Reid and Whitehill King [15] dubbed an *assurance appeal*, which intends to inspire trust and confidence in the procedure and/or physician. Textual elements reinforce this credibility and trustworthiness of the clinics’ medical professionals by highlighting their experience and/or educational background. For example, the Harley Medical Group included a series of posts in 2019 in which they introduced their cosmetic surgeons by listing their educational attainments and Linia posted the CVs of some of their surgeons (March 19, 2018). The emphasis on the credentials of these medical professionals functions as a quality marker and is increasingly important as it denotes legitimacy and trustworthiness in a market fraught with issues and scandals, particularly in the UK where the government only recently announced that it will introduce a ‘licensing regime’ for noninvasive cosmetic procedures [12].

Alongside the presentation of medical professionals, a few of the posts by Dutch and the UK clinics (5% [*n* = 28] and 2%, [*n* = 5], respectively) included depictions of the medical environment in which procedures take place or incorporated snapshots of the performance of cosmetic procedures^3^. Reflecting Instagram’s affordances and the platforms’ encouragement to share snapshots of people’s daily lives, these visual depictions in the Instagram posts—albeit rare—contrast with previous traditional marketing representations which bypassed the reality of procedures, highlighting easy transformations instead [14, 20].

In addition to visual depictions of procedures and an emphasis on qualified and experienced medical personnel, detailed information regarding the method and/or physicality of cosmetic procedures was available in 10 %[*n* = 40] of the Dutch and 7% [*n* = 26] of the UK data. A post by Linia, for example, explains what dermal fillers are and how they work; “they plump your skin, improve collagen production, and bring moisture to the area, literally ‘filling’ in those marionette lines” (May 22, 2019). In addition to describing or showing what procedures entail, approximately 7% [*n* = 44] of all clinics displayed and/or provided information regarding the specific products or instruments used during a particular procedure, such as various laser devices (e.g., Alizonne January 31, 2018; Kliniek Veldhoven January 23, 2019); syringes used in dermal filler treatments (e.g., Linia April 4, 2018; Faceland May 2, 2019); or implants used in breast surgery (e.g., Aklinieken April 7, 2018).

Despite descriptions of what procedures entail, and in line with previous research, less than 1% [*n* = 1] of the Dutch posts and 3% [*n* = 9] of the UK posts referred to the potential side effects or recovery associated with the procedures on offer. As Hermans (2021) has argued, the low prevalence of references to side effects and/or recovery can be partly explained in light of the pervasiveness of noninvasive procedures which generally carry less risk and require little recovery (time). However, noninvasive procedures are never risk-free; recognizing this, the Advertising Standards Authority prohibits “irresponsibly describing cosmetic interventions as ‘safe’ or ‘easy’, because it is likely that all interventions will carry some level of risk to the patient” [8]. Moreover, as of October 2018 all Dutch advertising for cosmetic procedures must carry the following warning: “Careful: beautifying yourself can turn [out] ugly. A successful procedure starts with a suitable doctor” [my translation] [10]. Nevertheless, out of the 260 posts by Dutch clinics published after October 2018, only one emphasized the importance of selecting a “suitable doctor” (Velthuis Kliniek, March 15, 2019), and two included an abbreviated version of the slogan (Van Rosmalen, March 9, 2019; April 10, 2019).

### Commercialism

While a small number of posts by cosmetic clinics discussed the medical nature and reality of cosmetic procedures, more commonly they revolved around commercial appeals related to appearance enhancement; positive psychological outcomes; self-determination; and rational aspects related to the clinic and procedures [15]. Moreover, posts often adopted a lighthearted tone, employing humor and/or particular emojis. These themes are explored in-depth in the following subsections.

#### Empowering You to Look Better and “Feel Fabulous”

In line with cosmetic practitioners’ aim to enhance bodily features “to improve appearance and confidence” [40], the clinics’ Instagram posts visually and textually emphasized how particular procedures would improve appearance, which would contribute to recipients’ increased psychological well-being. When sharing user-generated content by recipients of cosmetic procedures, for example, clinics used hashtags such as #perfectbody and #bodygoals (Linia, March 4, 2018), and most UK clinics added captions framing these people’s bodies as beautiful and desirable:

1. “How **great** does our client [name] **look** after her Breast Augmentation” (Medical Group March 1, 2019; April 2, 2019; March 6, 2019; June 10, 2019)
2. “[Name] breast enlargement done with us look how **amazing** she **looks”** [*emojis: face screaming in excitement; raising hands; flushed face; & heart eyes*] (Linia, March 4, 2018)
3. “[*Emoji: heart eyes*] [Name] looks **fabulous** 2 years post op with us” (Transform Hospital Group, July 5, 2018)

Though Dutch clinics also posted (before and after) images of recipients of cosmetic procedures, they did not include captions which reinforced particular beauty ideals or standards. Rather, when recipients were shown, the caption either did not refer to the image or it consisted of a quote by recipients, meaning that the clinics did not frame a person’s appearance or story. Nevertheless, the mere (re)posting of a before/after image may still signal the desirability of a cosmetically altered appearance. The lack of explicit positive evaluations of a model’s appearance by Dutch clinics may relate to Dutch advertising regulations which prohibit marketers from giving the impression that a particular body shape or specific physical characteristic is preferred [10].

In keeping with the wider appearance-enhancement industry which reinforces and capitalizes on seasonal body dissatisfaction [41], some of the clinics invoked discourses related to preparing bodies to be ‘summer-’ or ‘bikini-ready’. For example, 18% [*n* = 48] of the UK posts featured young women, often after breast augmentation procedures, in bikinis or other swimwear at a pool- or seaside^4^. The emphasis on achieving a body that is ‘summer-ready’—which, judging from the clinics’ posts includes full, shapely buttocks and breasts, and excludes any type of body hair and ‘excessive’ fat on the abdomen, hips and/or legs—was reflected in captions by both the Dutch and UK cosmetic clinics. Across the data, several clinics referred to (creating) ‘bikini’ bodies and urged people to prepare their bodies for being on display in summer (cf. Linia June 7, 2019; June 11, 2019; Transform August 3, 2018).

Alongside acquiring a physical ‘summer body’, some clinics emphasized that “confidence is key when you’re stepping into your bikini!” (Linia June 29, 2018). This link between appearance and mental well-being—albeit relatively uncommon in the data^5^—follows previous research which found that discourses around cosmetic procedures emphasize both physical and psychological benefits [6, 20]. However, whereas Hermans [6] found that cosmetic clinics were hesitant to draw explicit links between undergoing a procedure and improved confidence in light of the UK advertising regulations which stipulate that “marketers should not play on consumers’ insecurities [and] should not irresponsibly imply that a cosmetic intervention will be able to solve a consumer’s personal or emotional problems” [9], social media posts were less cautious. Linia, for example, promised that “having a blepharoplasty procedure increases your confidence and self-esteem” (March 1, 2018) and that “breast surgery can give you the confidence you’ve always wanted” (June 30, 2018). Similarly, Kliniek Veldhoven described hair transplantation surgery as “a confidence boost” (July 2, 2018, my translation).

In addition to clinics’ claims related to improved self-confidence, posts included first-person accounts by recipients, highlighting procedures’ beneficial impact on their self-esteem. Moreover, some clinics preferred to refer to (self) confidence more generally, without explicitly connecting it to cosmetic procedures; for example, the Van Rosmalen Kliniek asserted that they deem it essential “that everyone feels good about themselves” (January 16, 2019, my translation) and a post by MYA (January 25, 2018) asks followers, “what does confidence mean to you?”. Although these clinics do not explicitly link confidence to procedures, the context is important. The fact that these providers of cosmetic enhancements discuss confidence is significant, as becomes clear in people’s comments to MYA’s post: feeling confident was equated with the breast augmentation procedures women wished to undergo or had already undergone.

Underlying the clinics’ discourses of looking good and improving confidence are self-determination appeals which draw on neoliberal ideology as they encourage people to take control of their lives and undergo cosmetic procedures for their own benefit. As Hennink-Kaminski et al. (2010) and Harris-Moore (2014) found, these appeals have become increasingly popular in mass media and marketing where recipients of cosmetic procedures are constructed as active agents who see the decision to undergo procedures as a marker of self-care, an act of individual empowerment where they take control over their bodies’ appearance [42]. Transform Hospital Group employed this discourse of empowerment by adding stickers with the text “do it for you” to its Instagram posts between February 2018 and February 2019. The emphasis on (bodily) autonomy may also constitute a reaction against (feminist) concerns with and critiques of the cosmetic industry and, at times, recipients of cosmetic procedures [44–46]. MYA, for example, stated the following:

“We understand that cosmetic surgery is, to some, a controversial subject and certainly a misunderstood one. With that can come prejudice and in the extreme, a desire to ban cosmetic surgery. If you do not believe that adult women should be able to freely choose to have cosmetic surgery then you will always view our service negatively” (July 13, 2018)

**Fig. 2:**
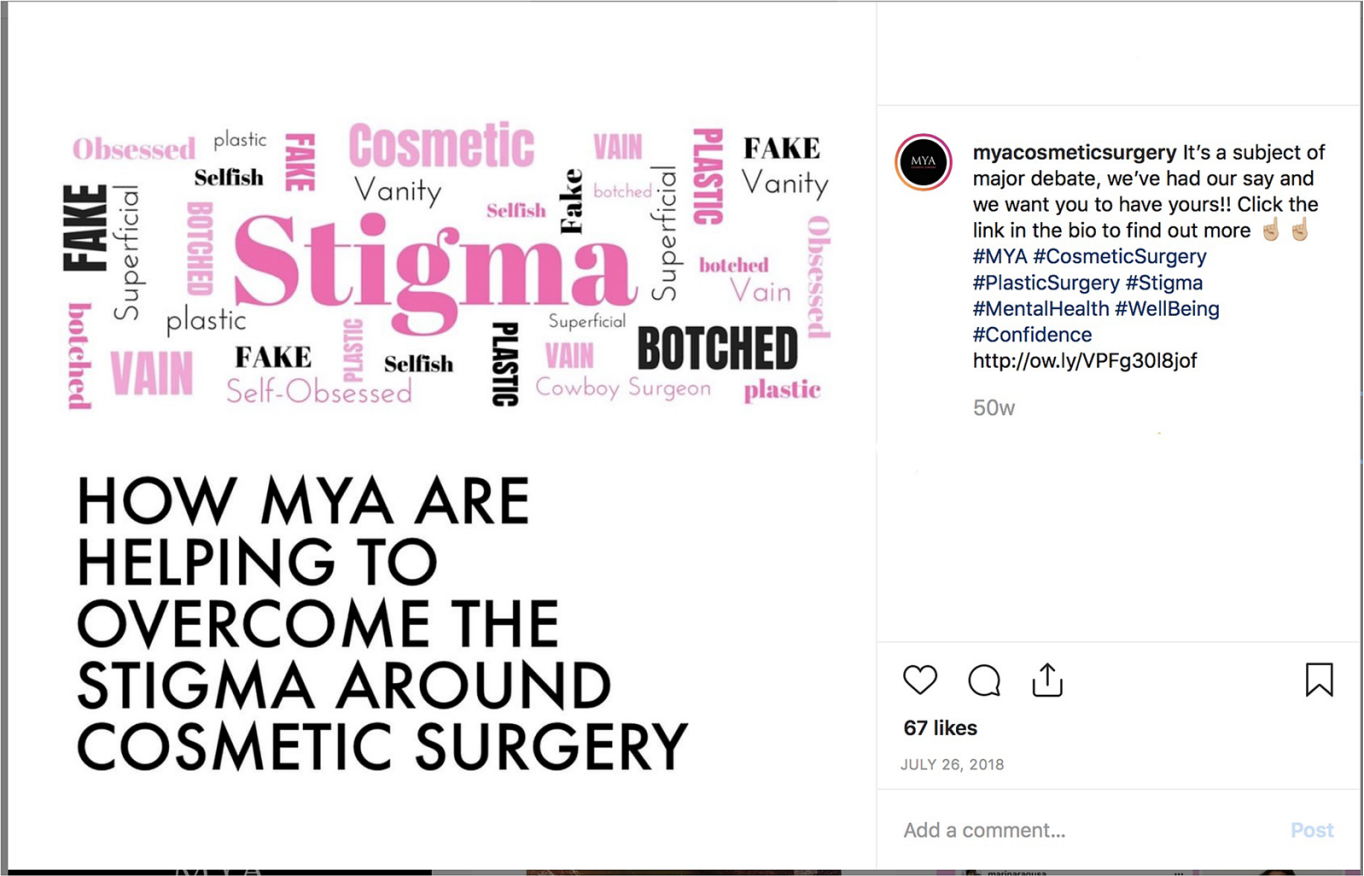
MYA July 26, 2018

Challenging some of the negative perceptions and/or prejudices regarding (recipients of) cosmetic procedures, MYA also illustrated various stereotypes and stigmas through a word cloud (see Fig. 2). Echoing concerns related to the presupposed selfishness and/or vanity of its clients, MYA argued that “falling in love with yourself first doesn’t make you vain or selfish, it makes you indestructible” (July 14, 2018). It is unclear here, however, whether this ‘falling in love with yourself’ would require—or would somehow legitimize—a cosmetic procedure.

Discourses related to curating a positive body image are present across marketing efforts within the beauty industry [46] and are reinforced by current body positivity and neutrality movements which have been popularized on social media platforms [37]. Although it is not this paper’s aim to assess whether undergoing cosmetic procedures can constitute an act of empowerment, it must be noted that actions are never independent of the societal context in which they take place, i.e., the current neoliberal, consumerist climate which conceptualizes bodies as malleable projects and which places a premium on beauty [42, 43].

#### Selling Cosmetic Procedures as Affordable, Quick, and Easy

Alongside marketing messages related to the psychological and/or physical (appearance) benefits which cosmetic procedures may entail, some of the clinics’ posts included information regarding finance and/or the quick and easy nature of procedures.

As cosmetic procedures are rarely covered by public healthcare systems, people must pay for procedures themselves; consequently, price plays an important role [24]. Nevertheless, cosmetic clinics seemed hesitant to include financial information in their posts; only 9% [*n* = 36] of the Dutch and 11% [*n* = 34] of the UK clinics included information regarding the financial aspects related to cosmetic procedures in their Instagram posts [15]. Most references to finance, particularly in the UK data^6^, presented specific offers, introduced special deals on noninvasive procedures (e.g., Linia May 1, 2018; Transform July 10, 2018), or offered discount packages when bringing a friend (Faceland May 17, 2019). In addition to these special offers, 3% [*n* = 13] of the Dutch and 2% [*n* = 7] of the UK posts promoted a giveaway or competition where people could win vouchers or non-surgical treatments by sharing selfies, tagging friends, liking and/or commenting on posts, or subscribing to the clinic’s newsletters.

There are several explanations for the relatively low number of references to financial aspects in the data, despite audiences indicating to find this information important in advertising for cosmetic procedures [24]. Firstly, the inclusion of pricing information and offers may transform medical professionals into salespeople which may negatively impact practitioners’ professional reputation and could undermine the integrity of the specialty. Moreover, although advertising authorities in the UK and the Netherlands allow clinics to market offers which are not time-sensitive, they do warn against loyalty schemes or ‘refer a friend’ incentives and emphasize that offers cannot “encourage consumers to undergo unnecessary or unwanted interventions” [8].

Alongside a commercial focus on price, some of the clinics emphasized the minimally invasive nature of the procedures they offered and/or the swiftness of the procedure or outcome^7^. Particularly one of the Dutch clinics advertised that some of their noninvasive procedures were “completely painless”, would “only take 30 minutes” and “required no recovery time”, which means people could “immediately resume their daily activities” (Aklinieken March 16, 2018; February 9, 2019; March 18, 2019, my translation). In addition to noninvasive procedures, eyelid surgery was also described as a “relatively simple and safe procedure” (April 9, 2019; March 12, 2019, my translation). Despite the emphasis on *relative* simplicity and safety, statements such as these may trivialize cosmetic procedures, which advertising regulations warn against.

#### Lighthearted Tone—Humor and Emojis

Social media affordances allow brands and organizations to establish and nurture more personable relationships with (prospective) clients and/or patients [47]; for example, the UK and Dutch clinics employed a variety of inspirational and/or funny quotes in their marketing. Moreover, reflecting general marketing strategies, clinics capitalized on a wide variety of holidays, from widely celebrated (Christian) holidays like Christmas to International Women’s Day (8^th^ of March) and even ‘Warm Sweater Day’ (15^th^ of February). Interestingly, reflecting higher appearance engagement and pressures among women, a discrepancy was found in Father’s Day and Mother’s Day offers. Whereas several Dutch and the UK clinics encouraged their followers to “say ‘thank you mum’ with our Mother’s Day packages” (Linia, March 11^th^, 2018), Father’s Day posts generally merely invited people to “tag your dad and tell us why he is the nicest and best dad” (Faceland June 14^th^, 2018, my translation).

In addition to posts related to holidays and inspirational and/or humorous quotes, the cosmetic clinics also made frequent use of emojis; 30% [*n* = 119] of the Dutch and 55% [*n* = 167] of the UK posts included at least one emoji.

As can be seen in Table 2, emojis expressing enthusiasm and/or appreciation were most common in both the Dutch and UK posts; through hearts, stars, and raising hands, the clinics demonstrated and evoked excitement about particular treatments, offers, or—most commonly—treatment outcomes. Of interest here is the use of fruit emojis to signify body parts—i.e., peaches symbolize buttocks, whereas coconuts and watermelons indicate (women’s) breasts. Whereas only one post by one Dutch clinic used a peach emoji to signify buttocks, these fruit emojis were more popular in the posts by the UK clinics. The use of these emojis, which are inherently lighthearted and colloquial and are indicators of ‘healthcare commercialism’ [4], raises some concerns related to the framing and target audience of these posts. After all, emojis make clinics’ content more relatable and more relevant to a younger target audience [48]. In light of the medical nature of the procedures that these clinics are offering, the use of emojis thus needs to be approached with caution.

**Table 2:** Overview of prevalence emojis in Dutch and the UK clinics

Category emoji	Emoji	Dutch clinics	The UK clinics	Total
Enthusiasm/appreciation	Heart	37	87	124
	Heart eyes	17	34	51
	Star	32	28	60
	Star eyes	3	3	6
	Raising hands	4	9	13
	Total	**93**	**161**	**254**
Beach/summer	Sun	10	10	20
	Bikini	3	16	19
	Total	**13**	**26**	**39**
Body parts	Lips	9	10	19
Fruit for body parts	Peach	1	2	3
	Coconut	–	3	3
	Watermelon	–	8	8
	Total	**10**	**23**	**33**
Medical	Bandage	2	–	2
	Doctor	3	7	10
	Facemask	3	–	3
	Syringe	12	9	21
	Total	20	16	36
Total		136	226	362

As posts could employ more than one emoji, the total number of emojis is higher than the number of posts including emojis

## Conclusion and Implications

In light of current concerns related to the ethical marketing of cosmetic procedures [26, 49], the aim of this paper was to explore the question of how cosmetic clinics in the Netherlands and the UK present themselves on Instagram. Similar to previous work which adopted a positioning theory framework [4], it became evident that cosmetic clinics may shift between different positions and discourses, such as the medical-professional and the commercial, and a formal vs informal tone. Future research can replicate this study to assess these outcomes in different geographical and legislative areas.

Considering the implications of this study, several points can be made. Firstly, despite calls for ‘ethical’ marketing on social media—conceptualized as being expert-driven and educational in nature [50]—this study illustrates that marketing materials are not always in-line with current advertising guidelines. Whereas Dutch and UK advertising standards authorities warn against the trivialization of cosmetic procedures, arguing that marketing materials should advance the ‘rational use of procedures’, and that clinics cannot suggest that procedures should be undertaken lightly, and/or are without risk, many posts by clinics backgrounded the medical nature of procedures. Clinics rarely mentioned risks and potential side effects; rather some clinics, particularly in the UK, presented aspirational holiday-visuals of young women in bikinis, utilized emojis to engage (younger) audiences, or posted inspirational or funny quotes.

A second concern relates to the presentation of a narrow beauty ideal by the clinics, which has been shown to negatively impact people’s body perception and self-image [29]. Although the Dutch advertising Code prescribes that marketing materials for cosmetic procedures cannot give the impression that a particular body shape or specific physical characteristic is preferred, the models in the clinics’ Instagram post lacked diversity; most were young, white women with an ‘appropriate’ weight. Moreover, models’ bodies which diverged from this norm—i.e., when they were ‘larger’—were problematized and medicalized as the clinics offered various (non-) invasive procedures to lose weight.

In conclusion, the marketing by cosmetic clinics on social media is not always in-line with advertising guidelines. As marketing on social media is difficult to monitor and regulate [50], and the industry also relies on self-regulation, it is important that we gain an understanding of the reasons why medical cosmetic clinics do not adhere to guidelines, as this may be due to either ignorance or a willful disregard of the rules. Depending on the outcomes, it may be necessary to reassess the dissemination of advertising guidelines and/or the establishment of clear sanctions.

## Footnotes

1 This is also manifest in the fact that cosmetic procedures are generally not covered by health insurance (NL) or the national health service (UK). There are exceptions to this; for example, rhinoplasties may be indicated when a person experiences issues breathing, and eyelid corrections may be required in severe cases of ptosis which result in impaired vision. However, the question here is whether these procedures can be considered ‘cosmetic’ or whether these are more ‘reconstructive’. For a more extensive discussion on the conceptualization of plastic, reconstructive, and cosmetic surgery, see Gilman [51] and Hermans [6].

2 Although this scale only adopts rather crude illustrations of the human form and inadequately considers ethnic features (Jayawardena et al., 2021), this simplified overview of different body shapes sufficed for the purposes of this research.

3 The Dutch clinics included significantly more visuals of procedures [7% of all posts, *n* = 28] than the UK clinics [2% of all posts, *n* = 5] (χ^2^(1, 1)=11.44, *p* < 0.001).

4 Comparatively, only 4% [*n* = 16] of the Dutch posts showed women in swimwear at a pool- or seaside; this difference between the UK and the Netherlands was statistically significant (χ^2^(1, 1)=28.14, *p *< 0.001).

5 References to confidence and feeling good are present in 5% [*n* = 20] of the Dutch posts and in 9% [*n* = 28] of the UK posts. Similar to discourses of looking good being more prevalent in the UK data (χ^2^(1, 1)=34.82, *p *< .001), the UK clinics also included significantly more references to feeling good and confidence than their Dutch counterparts (χ^2^(1, 1)=4.52, *p*=.034).

6 91% [*n* = 31] of the finance references in the UK posts and 47% [*n* = 17] of those in the Dutch posts related to specific offers.

7 7% [*n* = 28] of the posts by Dutch clinics and 2% [*n* = 7] of the UK clinics referred to the minimally invasive nature of procedures and/or the quick nature of procedures or outcomes.
